# My appendectomy

**DOI:** 10.3402/jchimp.v1i4.10847

**Published:** 2012-01-26

**Authors:** Malek Cheikh

**Affiliations:** Union Memorial Hospital, Baltimore, MD, USA

## Abstract

A personal experience of a medical resident with appendicitis

As an internal medicine third-year resident, I recently switched roles and became a hospitalized patient. My symptoms started 3 months ago when I was working with one of my mentors in his office. That morning I awoke with generalized abdominal pain. I thought it would subside in an hour or so, as I often have abdominal cramps.

However, it did not subside. As I drove to the hospital, I felt that my pain was increasing. With every bump in the road, the pain felt like twisting in my guts! Eventually I arrived at our grand rounds, for a talk on one of the subjects of which I am fond of, ‘Subclinical Thyroid Disease.’ I could not concentrate because my abdominal pain was severe enough that I had to reposition myself every 20 seconds in the chair and I was sweating profusely. Until that point, I had not thought of my appendix. I was 27, never had surgery in my life nor a family history of any surgery. I considered myself generally healthy.

I went to the office where I was supposed to be. Immediately after seeing the first patient, I knew that I was not going to make it until the end of the day because the pain was too severe. I told my attender that I needed to go home. When I told him about my ‘bumpy ride,’ he recommended that I see a surgeon. I resisted at first, but when the pain started to localize in my right lower quadrant, I knew I should stop procrastinating.

Labs and a computerized tomography (CT) scan were done and showed a WBC of 16,000 and radiological findings compatible with acute appendicitis (1). However, by the time we had all this information, I was feeling better and the pain had greatly subsided. Therefore, I made the decision that I was not going through with the surgery, at least for the time being. I followed my internal medicine gut and applied the well-known medicine axiom, ‘wait and see.’

It was 3 months before the pain returned. This time, the pain came back in different forms. It started when I was driving back from a friend's house, (incidentally, this friend happened to be a surgeon working with me in the same hospital, and I still blame him for my surgery). When the pain reoccurred this time, it was severe nausea; I vomited eight times, with no relief. Soon, the abdominal pain generalized with a positive McBurney's sign. That was enough for me to wake up my wife and call my brother for a lift to my hospital's emergency room (ER).

The ER attender reviewed my previous information and at this time my WBC was 15,000. They started hydrating me. The surgeon who originally saw me was on call and the decision to take me to undergo an appendectomy was made on the basis of clinical suspicion without repeating the abdominal CT.

My operation lasted 50 min. I had an open appendectomy that left me with a one inch surgical incision and three staples. The surgeon told me that the appendix had scars pointing toward a chronic inflammation, but the pathology report came back positive for acute appendicitis with no chronic component. I was debating whether I should keep my appendix preserved in a jar, but when I went down to the pathology lab, it did not look precious enough to keep.

**Figure:**
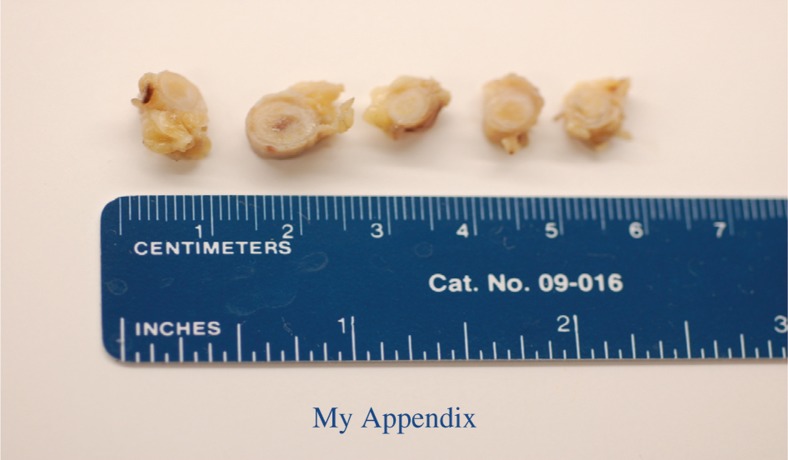


I came across an article in the New York Times about a physician who gets to experience being a patient once. He said ‘If anything, it's that recognition of vulnerability as well as expertise that makes me a better doctor today’ (2). My experience was not as dramatic as his was, but it made me see and feel things I was not able to appreciate as a doctor. The only way to recognize them was to experience them in a patient's shoes (or socks). And, now my myopia has cleared and I am more empathetic about the patient's experience.

Lessons learned from my hospitalization are listed below:

1. Subcutaneous heparin burns!!!
2. I always thought that IV fluids are harmless in a patient with good liver, heart, and kidney function, but not after having many liters of fluids running through my veins post operation when I was unable to urinate or even walk to the bathroom without getting pain from the surgical site.
3. Nurses are angels!!! They were so considerate and caring; much different than how they interact with residents.
4. Always talk to your patients, especially to inform them that you are changing their pain medications. It is frustrating to have your needs ignored.
5. It was exhausting. I was amazed at how a one night hospital stay can drain a person's energy. There was not a time where I had more than 10 min rest. There was always someone coming in to change the fluid bag, check vitals, or give pills.
6. Patients have the right to be angry, frustrated, and depressed. At some point of my hospitalization; I felt all of the above.
7. Hydromorphone can make all your troubles go away (for about 4–6 hours)!!!
8. Never turn your back on a surgical resident; they will pull a prank on you!! Sending a student to perform a manual disimpaction when you are not constipated is not funny.
9. Knocking on the door can save you and your patient (who may be using the urinal while sitting on the edge of the bed) a lot of embarrassment, especially if you and your patient are colleagues working in the same hospital.
10. No matter how fashionable and trendy you dress in real life, the hospital gown will take that away from you and bring you down to earth!!!
